# Durability, Strength, and Erosion Resistance Assessment of Lignin Biopolymer Treated Soil

**DOI:** 10.3390/polym15061556

**Published:** 2023-03-21

**Authors:** Pouyan Bagheri, Ivan Gratchev, Suwon Son, Maksym Rybachuk

**Affiliations:** 1 School of Engineering and Built Environment, Griffith University, Engineering Drive, Southport, QLD 4222, Australia; 2 Department of Architectural and Civil Engineering, Kyungil University, Gyeongsan 38428, Republic of Korea; 3 School of Engineering and Built Environment, Griffith University, 170 Kessels Rd., Nathan, QLD 4111, Australia; 4 Centre for Quantum Dynamics and Australian Attosecond Science Facility, Griffith University, Science Road, Nathan, QLD 4111, Australia

**Keywords:** lignin biopolymer, erosion, soil strength, triaxial test, wetting and drying cycles, silt

## Abstract

To mitigate the negative environmental effects of the overuse of conventional materials—such as cement—in soil improvement, sustainable engineering techniques need to be applied. The use of biopolymers as an alternative, environmentally friendly solution has received a great deal of attention recently. The application of lignin, a sustainable and ecofriendly biobased adhesive, to enhance soil mechanical properties has been investigated. The changes to engineering properties of lignin-infused soil relative to a lignin addition to soil at 0.5, 1, and 3.0 wt.% (including Atterberg limits, unconfined compression strength, consolidated undrained triaxial characteristics, and mechanical properties under wetting and drying cycles that mimic atmospheric conditions) have been studied. Our findings reveal that the soil’s physical and strength characteristics, including unconfined compressive strength and soil cohesion, were improved by adding lignin through the aggregated soil particle process. While the internal friction angle of the soil was slightly decreased, the lignin additive significantly increased soil cohesion; the addition of 3% lignin to the soil doubled the soil’s compressive strength and cohesion. Lignin-treated samples experienced less strength loss during wetting and drying cycles. After six repeated wetting and drying cycles, the strength of the 3% lignin-treated sample was twice that of the untreated sample. Soil treated with 3% lignin displayed the highest erosion resistance and minimal soil mass loss of ca. 10% under emulated atmospheric conditions. This study offers useful insights into the utilization of lignin biopolymer in practical engineering applications, such as road stabilization, slope reinforcement, and erosion prevention.

## 1. Introduction

Global climate change has dramatically influenced the environment, leading to irreversible changes in the limited resources we rely on. This has urged fundamental sustainable measures to be taken to reduce the consequences of these effects. A major impact of climate change that is significantly affecting the environment around us is extreme weather events, which results in intense localized rainfall in some geographic areas and drought in others. Intense rainfall events may cause instability in the ground properties, bringing a sudden increase in pore water pressure in soil which incurs a reduction in local soil strength, severe runoff, soil erosion, and eventually landslides and slope failures.

To improve the mechanical properties of soil and soil stability, a range of chemical treatments, including the addition of stabilizers to the soil, are often used. Portland cement has traditionally been the most commonly used additive to enhance soil properties. Although Portland cement has been widely used in different geotechnical engineering practices, its application for soil enhancement has been associated with a negative impact on the environment, alongside the increase of carbon dioxide (CO_2_) emissions during cement production, as cement industries are believed to be responsible for up to 8% of global CO_2_ emissions annually [1]. The application of cement for civil and geotechnical engineering purposes is believed to have contributed to several environmental concerns, including the increase in soil pH level [2], cement dust accumulation in soil resulting in soil infertility [3], urban runoff, heat islands, prevention of vegetation growth, and groundwater contamination [4].

Recently, research has been undertaken to use biopolymers, such as organic polymers that occur in abundance in nature and can be extracted from natural resources, as environmentally friendly additives in geotechnical engineering applications [5].

The improvement of soil strength by adding biopolymers, such as xanthan gum, guar gum, beta glucan, chitosan, and lignin, has been attempted before [4,5,6,7,8,9,10,11,12,13]. Bagheri et al. [5] examined the effect of xanthan gum on soil strength and confirmed substantial improvement in soil compressive strength within a certain curing time. Through lab studies, Soldo et al. [4] investigated the impact of xanthan gum, beta glucans, guar gum, chitosan, and alginate biopolymers on silty sand soil. They reported significant increases in biopolymer-treated soil strengths over a longer period. The effectiveness of gellan gum biopolymer against soil permeability [14] and dextran for surface erosion [13] were investigated. Ham et al. [13] added a microbial biopolymer, dextran, to the fine silica sand and showed that the biopolymer can increase erosion resistance. Zhang et al. [15] performed the shear-wave velocity test and unconfined compression test to assess the small-strain shear modulus and unconfined compressive strength of lignin-stabilised silty soil. They found that a small-strain shear modulus and the unconfined compressive strength of lignin-treated soil logarithmically increased with curing period.

To increase the effectiveness of biopolymer treatment, in situ influencing factors must be taken into consideration. Although lignin has been shown to boost soil strength [10,15], prior research largely focused on analyzing basic strengthening behavior and confirming viability. In particular, in situ three-dimensional stress conditions have not received significant consideration. In addition, studies that address changes in mechanical strength of lignin-treated soils under saturated conditions have been limited, including those concerned with the erosion resistance of biopolymer-treated soil. Furthermore, the earlier reports mostly evaluated the shear behaviour of biopolymer-treated soils by means of direct shear tests using dried soil samples that may not adequately represent the underground conditions.

Lignin was chosen for use in the current investigation because it has been demonstrated to be one of the most economically advantageous materials among all available biopolymers in geotechnical engineering [16].

This study aims to address the mentioned gaps by performing a thorough study to examine the effect of lignin biopolymer agent on the soil mechanical properties subjected to various conditions. A range of laboratory experiments, including Atterberg limits tests and unconfined compressive strength (UCS) tests for the lignin-treated soil samples were conducted to obtain the engineering performance, soil strength, and plasticity behaviour. To investigate the effect of lignin additive on the soil shear strength and shear parameters and simulate in situ three-dimensional stress conditions, CU triaxial tests were performed. The durability and strength of lignin-treated soil under wetting and drying cycles were examined. The erosion resistance and soil loss of biopolymer-treated specimens exposed to natural atmospheric conditions were also examined. Finally, scanning electron microscopy (SEM) analyses were conducted to evaluate the microstructure mechanism of such treatment approaches.

The outcome of this study provides an enhanced understanding of the engineering behaviour of lignin-treated soil subjected to different conditions and facilitates the use of such sustainable techniques in civil and geotechnical practices.

## 2. Materials and Methods

### 2.1. Materials

**Figure 1:**
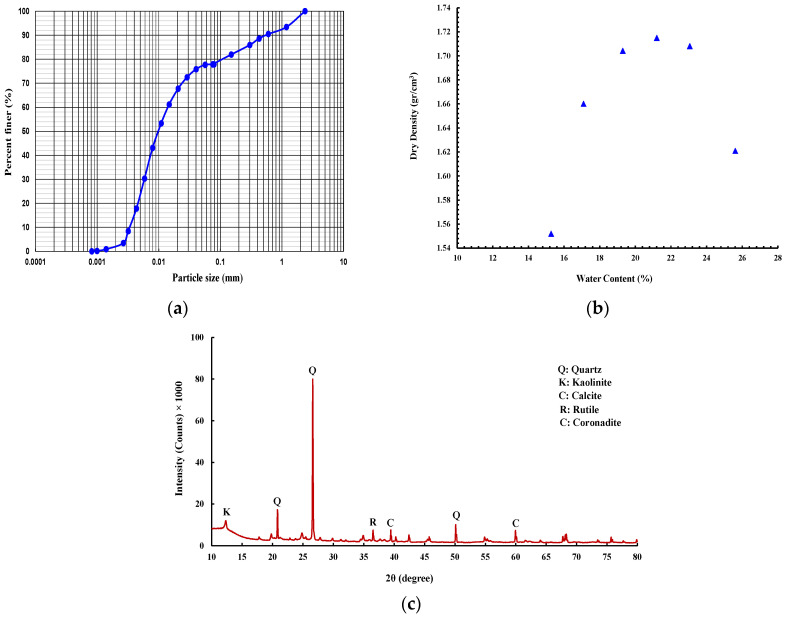
(**a**) Grain size distribution. (**b**) Compaction test result. (**c**) X-ray diffraction (XRD) patterns of soil.

Regarding soil, a low-plasticity silt soil (USCS classification: ML) according to ASTM D2487-17 [17] has been obtained from the Gold Coast area, Australia, with the grain size distribution as shown in Figure 1a obtained following the ASTM D422-63 [18]. The soil sample displayed plastic and liquid limits and a plasticity index of 26.9, 38, and 11.1, respectively, as measured in accordance with ASTM D4318-17 [19]. The specific gravity of the soil was 2.77, according to the ASTM D854-14 [20]. The standard proctor compaction test following ASTM D698-12 [21] was performed to obtain the maximum dry density of (1.72 g/cm^3^) and optimum moisture content of (21.7%) (Figure 1b).

The mineral compositions of the soil were supplied by X-ray diffraction (XRD) analysis (Figure 1c). As seen from the soil XRD patterns, quartz is the main mineral with additional inclusions of kaolinite and calcite.

Regarding biopolymers, the lignin (LIG) was calcium lignosulphonate obtained from Dustex, Australia. The material was a brown viscose liquid with a pH (10% solution) of 5.4 ± 3.0, dry matter of 55.0 ± 1.0%, and a density of 1285 kg/m^3^. The LIG was a mixture of water (51%) and calcium lignosulfonate (49%).

### 2.2. Specimen Preparation

Initially, the soil was oven dried, and then the gravel was removed by crushing and sieving to 2.36 mm. Three concentrations of soil to LIG mixtures at 0.5 wt.%, 1.0 wt.%, and 3.0 wt.% were used in the study.

The wet mixture approach, as described by Ta’negonbadi et al. [10], was used to prepare LIG soil mixture. The LIG liquid was first added to the water to reach the desired moisture content, and then the diluted solution was sprayed and thoroughly mixed with the dry soil to prepare the homogenous blend.

The obtained mixtures were wrapped with double-layer plastic wrap and kept in a controlled temperature room for 24 h to prevent the formation of aggregations and ensure a uniform combination of biopolymer with soil particles. The soil mixtures were placed into a cylindrical metal mold (diameter 50 mm, length 150 mm) and evenly compacted in five layers to prepare the samples. The samples with the same diameter and length of around 110 mm were extruded from the mold following each compaction set. Each sample’s dry density was guaranteed to be greater than 95% of the maximum dry soil density.

### 2.3. Experimental Measurments

Atterberg limits tests were conducted for the untreated and specified concentrations of LIG-treated soil to evaluate the impact of the biopolymer additive on the soil plasticity.

Regarding UCS tests, the samples were cured in a controlled temperature room for 0, 1, 4, 7, 10, 14, 28, and 35 days. The UCS tests in accordance with [22] were carried out for the cured samples. It is worth noting that three samples for each test were tested to minimize errors. From the outcome of UCS tests, the optimum curing time for treated and untreated samples was chosen for the following experiments.

Triaxial tests were used to examine how saturation conditions affected the strength of soil treated with LIG biopolymer. Consolidated undrained (CU) triaxial tests for the saturated samples were performed at confining pressures of 50, 100, and 200 kPa.

Regarding wetting and drying cycles tests, the durability of biopolymer-treated samples over six wetting/drying cycles was investigated. Polyvinyl chloride (PVC) molds were constructed with a diameter approximately equal to the sample (51 mm) and a higher length of 130 mm, allowing the sample to expand during the wetting cycle. Each wetting and drying cycle was initially started by placing the cured sample into a PVC mold, and then the mold was submerged in water for 24 h. The sample was then dried under room temperature conditions for the given optimum curing time. The UCS test was conducted following each cycle’s completion to determine each sample’s compressive strength.

Regarding the field experiment, five samples, including two untreated and three treated with the given concentrations of LIG, were exposed to the environment for 30 consecutive days, and soil mass loss of each specimen after exposure to the atmospheric conditions was measured and calculated. Daily temperature, relative humidity, and rainfall were recorded to evaluate the effect of environmental conditions on each sample’s integrity.

Regarding SEM analysis, sample morphological examination was performed by using a scanning electron microscopy (SEM) analytical system (TESCAN Mira3, TESCAN Orsay Holdings, Czech Republic) under an acceleration voltage of 5 kV. Magnifications at 2500× and 15,000× were used and reported herein for the untreated, and 3% LIG-treated samples. The samples were platinum sputter coated (ca. 5 nm) immediately before collecting SEM image data. 

## 3. Results and Discussion

The following provide the results and thorough examinations of the impact of LIG biopolymer on the soil.

### 3.1. Atterberg Limits

The results of Atterberg limits tests considering different percentages of LIG are presented in Table 1.

**Figure 2:**
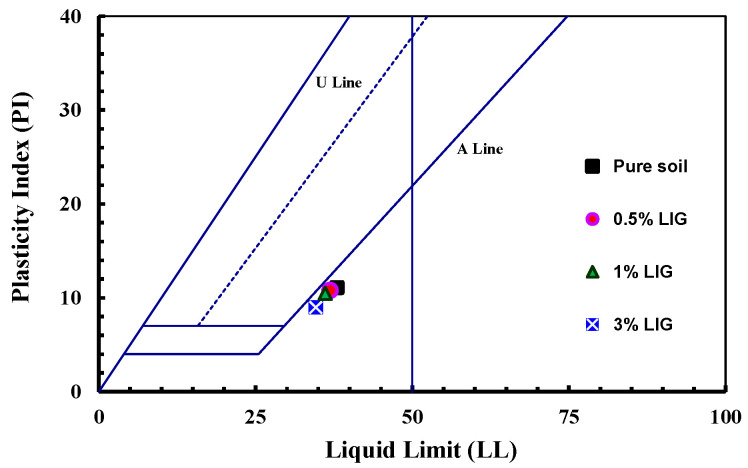
Plasticity chart for the untreated and LIG-treated soil.

The presence of LIG did not significantly affect PL, while the LIG-treated soil experienced a slight reduction in LL (Table 1). This can be related to the clay particles flocculation [23]. By increasing the content of LIG, the LL slightly decreased while the PL remained almost unchanged. As the soil has some negative charges from clay minerals, LIG may neutralize the negative charges on the surface of soil particles. This brings less thickness to the double electric layer between the soil particles [24]. All LIG-treated samples were placed below the A-Line in the plasticity chart, indicating a slight change in soil plasticity (Figure 2).

### 3.2. Unconfined Compressive Strength (UCS)

**Figure 3:**
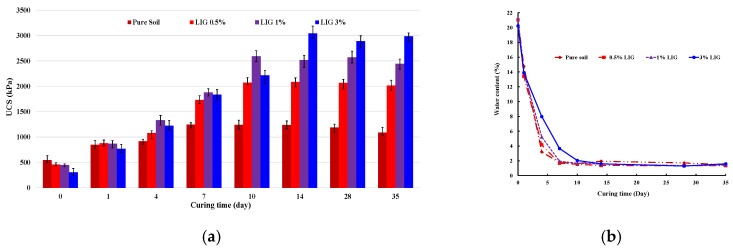
(**a**) Changes in UCS for the LIG-treated soil with curing time. (**b**) Variation of water content with curing time.

A series of UCS tests for the specified percentages of LIG-treated soils were conducted to ascertain the optimum curing time and analyze the compressive strength of the stabilized samples. Figure 3a displays the changes in UCS for the untreated and LIG-treated soils over various curing times.

Increased biopolymer concentration resulted in increased UCS. While the specimens treated with 0.5% and 1% LIG reached their peak strength after 10 days of curing, the 3% LIG-treated specimen reached its maximum strength after 14 days of curing. The UCS considerably increased within a certain curing time for all LIG concentrations. It demonstrates that additional curing time slightly affects the soil strength. Since moisture content has a significant impact on soil behaviour, the moisture content of each specimen at the end of UCS tests was measured and represented in Figure 3b. A significant drop in water content corresponded to a substantial increase in the soil strength (Figure 3b). When the treated samples dried, the LIG biopolymer acted like a glue leading to a noticeable increase in soil strength.

The curing time corresponding to the highest UCS was considered the optimum and used for the following tests.

### 3.3. Shear Strength

#### CU Triaxial

**Figure 4:**
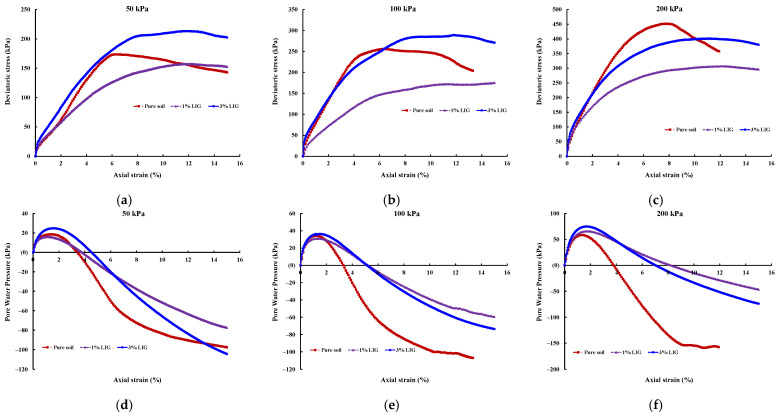
Results of CU triaxial tests, deviatoric stress-axial strain curves for (**a**) 50 kPa confining pressure; (**b**) 100 kPa confining pressure; (**c**) 200 kPa confining pressure; and pore water pressures for (**d**) 50 kPa confining pressure; (**e**) 100 kPa confining pressure; (**f**) 200 kPa confining pressure.

The effect of LIG biopolymer on the stress-strain curves and shearing-induced pore water pressures for the pure soil, LIG treated specimens are shown in Figure 4.

As shown in Figure 4, LIG causes a reduction in the brittleness of soil. Pure soil exhibited brittle behaviour, and peak deviator stress is clearly defined; however, there is no well-outlined peak of shear stress for LIG-treated samples.

The soil treated with 3% LIG experienced slightly higher strength than pure soil, especially at lower confining pressures (Figure 4a). An approximate 10% increase in peak deviator stress was observed once the soil was treated with 3% LIG at 50 kPa confining pressure. This relatively small strength enhancement is attributed to the increase in soil cohesion. In addition, the absolute values of pore water pressures developed upon shearing for soil treated with 3% LIG were marginally higher than the shearing-induced pore water pressures in pure soil (Figure 4d). This implies higher suction upon the shearing stage, which resulted in higher deviatoric stress in 3% LIG treated soil. The higher negative pressure causes an increase in effective stress applied to the soil particles and leads to increased soil shear strength.

**Figure 5:**
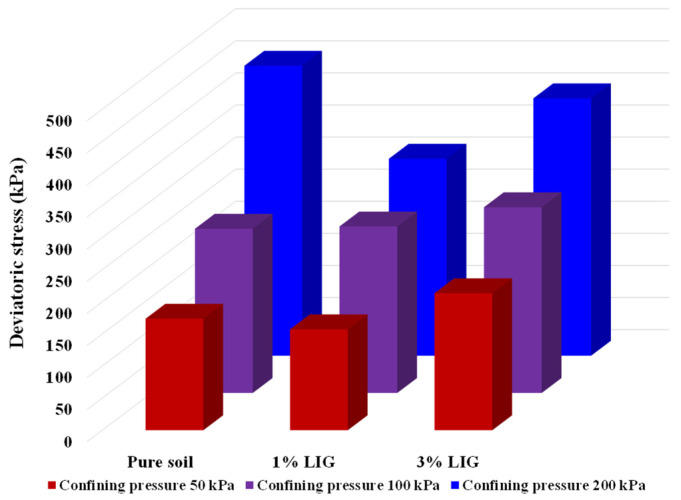
Results of CU triaxial tests, changes in maximum deviatoric stresses at various confining pressures.

To combine the obtained results of shearing of pure soil and LIG-treated soil at various confining pressures, the maximum deviatoric stresses are shown in Figure 5.

**Figure 6:**
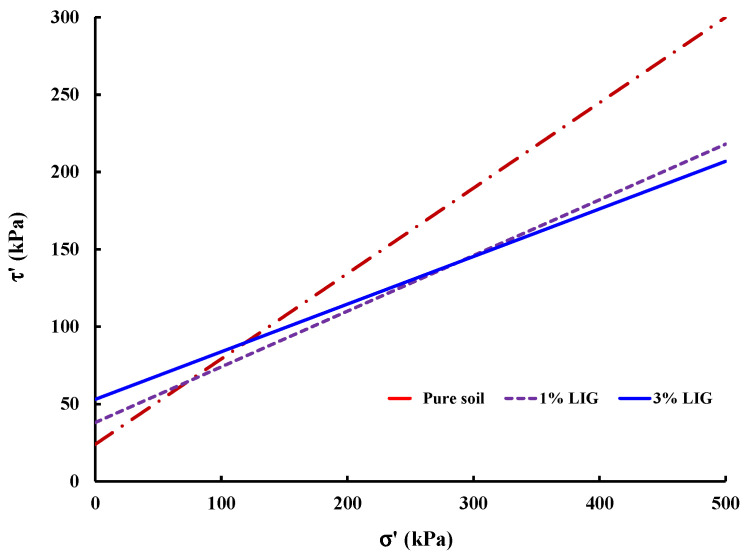
Effective shear stress vs. effective normal stress curves.

Plotted failure envelope curves were used to derive the soil shear parameters for untreated and LIG-treated soil (Figure 6).

While soil friction angle reduced to some extent, a significant increase in soil cohesion occurred due to the LIG additive. Typically, the angularity of soil particles, soil gradation, and normal stress affect the soil friction angle. The LIG additive covers soil particles, smoothening the surface of particles, which causes a reduction in soil grain angularity. This results in a reduction in overall friction angle of soil. Table 2 provides the calculated shear parameters.

### 3.4. Wetting and Drying Cycles

**Figure 7:**
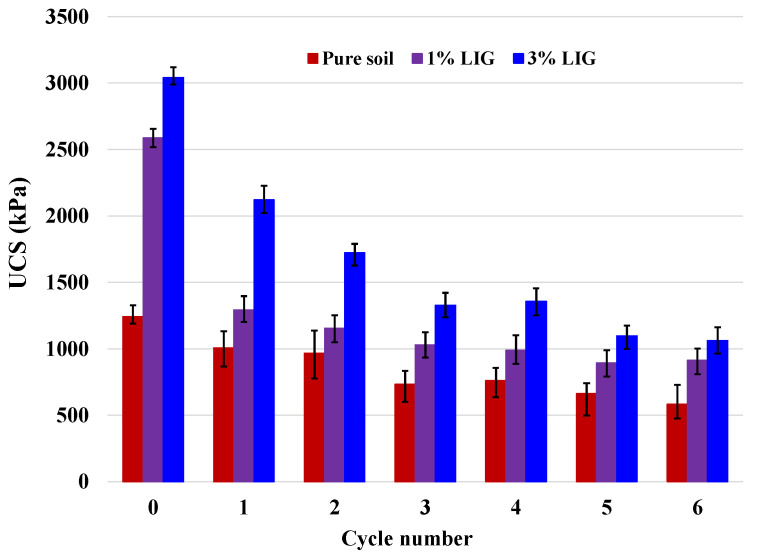
Changes of UCS with wetting and drying cycles.

Figure 7 shows the changes in UCS values over six wetting and drying cycles. As the number of cycles increased, the UCS of the untreated soil gradually dropped. Following a dramatic reduction in the second cycle, the soil strength for the 1% LIG-treated soil was slightly decreased by increasing the wetting and drying cycles. For the highest dosage of LIG, as the number of cycles increased, there was a noticeable decline in soil strength. To clarify the effect of wetting/drying cycles and to better understand the rate of strength reduction over each cycle, the reduction factor (*R_f_*) defined as Equation (1) and changes in *R_f_*, (∆*R_f_*), were computed and summarized in Table 3. We have

(1) $$R_{f_{i}}=\left(\frac{\left|S_{i}-S_{0}\right|}{S_{0}}\right)\times 100;\;\Delta R_{f}=R_{f_{i+1}}-R_{f_{i}},$$

where $S_{i}$ is the UCS after each cycle, and $S_{0}$ is the UCS at cycle 0 (before starting the experiment).

In all samples, the highest *R_f_* occurred during the first cycle. As seen after the second cycle, the ∆*R_f_* were relatively low, confirming that further wetting/drying cycles did not significantly affect the soil strength. The LIG-treated samples showed higher soil strength than the pure soil sample despite UCS reduction in all specimens. The reason is that after each wetting cycle, once the sample was exposed to the drying cycle, the LIG reattached soil particles and mended the cracks in samples, leading to stronger grain- bonding. This proves the capability of LIG biopolymer in reduction of soil strength loss in comparison with the untreated soil.

### 3.5. Exposure to Atmosphere Conditions

**Figure 8:**
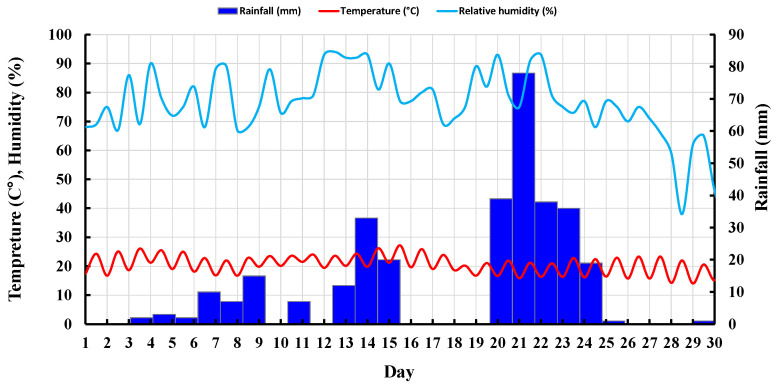
Recorded daily temperature, relative humidity, and rainfall in the field.

**Figure 9:**
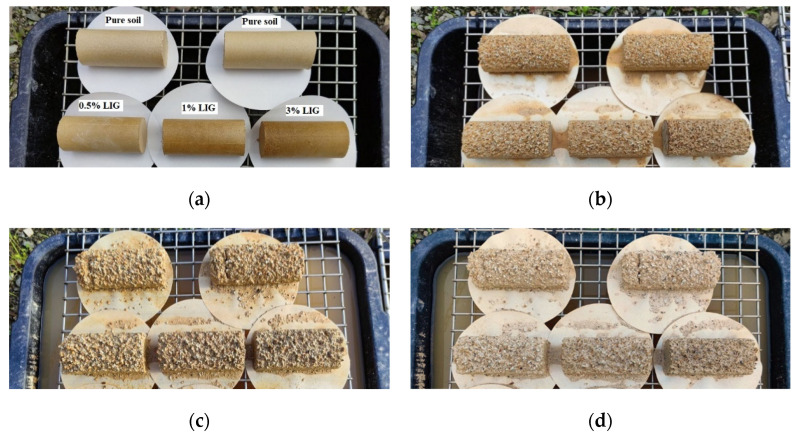
Samples exposed to the atmospheric conditions at (**a**) day 1, (**b**) day 14, (**c**) day 21, and (**d**) day 28.

**Figure 10:**
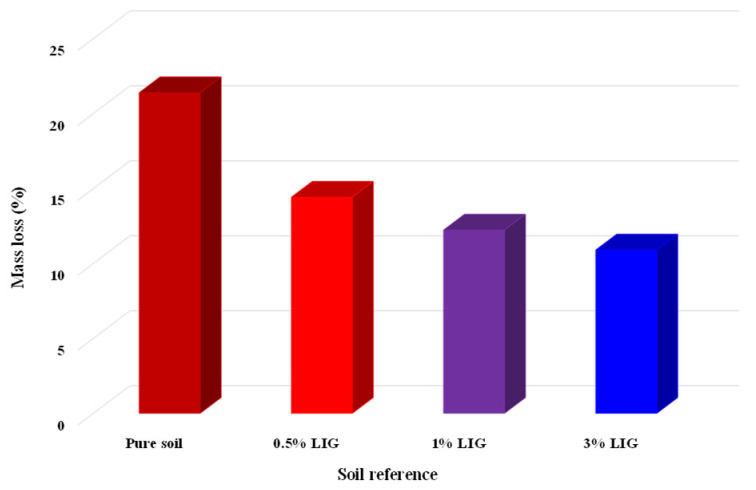
Soil mass loss after exposure to atmospheric conditions.

Five samples, including two untreated and three treated with the given concentrations of LIG, were first prepared and placed under atmospheric influences. Daily temperature, relative humidity, and rainfall in the field were recorded (Figure 8). Figure 9 shows the samples exposed to natural atmospheric conditions within 30 days of testing. Even though all samples lost some soil mass during rainfall events, they kept their shapes after two weeks of exposure to the atmospheric conditions. While the untreated samples were damaged and lost almost 22% of their soil masses, the LIG-treated samples kept their shapes. The 3% LIG-treated sample remained nearly intact after 30 days of atmospheric condition exposure. The soil mass loss after 30 days of atmospheric exposure is given in Figure 10. The LIG-treated samples lost less soil mass compared to pure soil samples, confirming the applicability of such treatment technique in improving soil erosion resistance.

### 3.6. SEM Analysis

**Figure 11:**
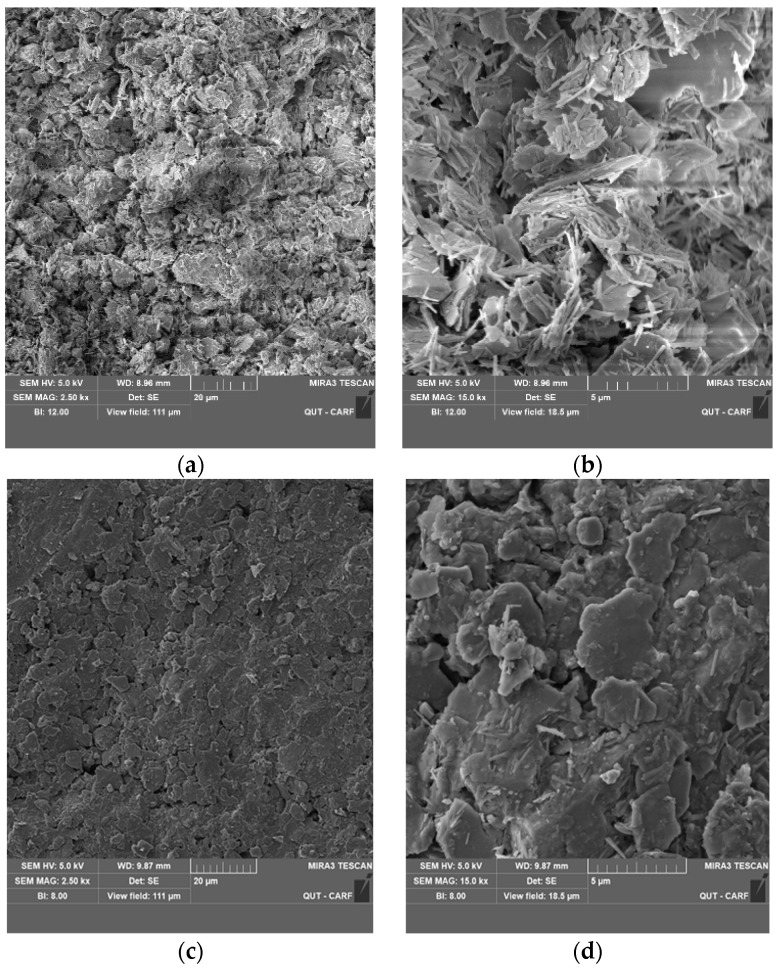
SEM images of the (**a**) pure soil with 2500× magnification; (**b**) untreated soil with 15,000× magnification; (**c**) LIG-treated soil with 2500× magnification; (**d**) LIG treated soil with 15,000× magnification.

SEM images of untreated and 3% LIG-treated soil are presented in Figure 11. The structure of soil changed due to lignin additive. The structure of pure soil has changed from a clumpy and grained-based form (Figure 11a,b) to a combined and more conjunct configuration (Figure 11c,d).

The structure of pure soil is uneven, flaky, and disjointed. There are more obvious chunks of soil particles with larger spaces (Figure 11a,b). However, in treated soil, the pores among soil particles are partially filled with LIG, and a clear biopolymer coating of soil particles’ surfaces and boundaries are seen. The soil particle aggregations by the LIG coating of the grains verify the soil strength improvement by adding biopolymer (Figure 11c,d).

## 4. Conclusions

The major goals of this study were to create an environmentally friendly biopolymer-based soil improvement method and to conduct an experimental investigation into the impact of LIG biopolymer on the mechanical properties and erosion resistance of soil. To achieve these goals, various laboratory and field experiments on three different concentrations of LIG soil mixtures were performed. The following represents a summary of the most significant findings.

- The effect of LIG additive appeared to be insignificant on the soil PL, and a slight reduction in LL and plasticity index was seen.
- The soil compressive strength significantly increased with increasing curing time and LIG dosage. After 14 days of curing, the compressive strength of 3% LIG-treated samples did not go under major changes, and further curing did not affect their strengths. A similar trend was seen for 0.5% and 1% LIG-treated specimens after 10 days of curing.
- At lower confining pressures of CU triaxial tests, the 3% LIG-treated soil experienced slightly higher shear strength than the pure soil. The LIG additive caused significant improvement in soil cohesion and a slight reduction in soil friction angle.
- Despite biopolymers susceptibility to water, during wetting/drying cycles, the LIG biopolymer showed its capability to improve the strength and durability of soil to water. While all samples experienced compressive strength reduction during wetting and drying cycles, the LIG treated samples showed significantly higher strength than pure soil.
- The LIG-treated samples lost less soil mass compared to pure soil samples confirming the applicability of such treatment technique in improving soil erosion resistance.
- This study showed that the LIG biopolymer, a byproduct of paper and sugarcane factories, can effectively improve soil strength and erosion resistance under various conditions. The durability of LIG additive to water during wetting/drying cycles proves its potential application for quick temporary constructions in an arid climate area. Additionally, lower soil mass loss of LIG-treated samples verifies its application in erosion-prone areas.
- As the durability of biopolymers against soil erosion is an important factor and needs further investigation, large-scale field tests should be linked in future studies.

## Figures and Tables

**Table 1:** Results of Atterberg limits tests.

Soil Reference	Liquid Limit, LL (%)	Plastic Limit, PL (%)	Plasticity Index, PI (%)
Pure soil	38.0	26.9	11.1
0.5% LIG	36.9	26.1	10.8
1% LIG	36.1	25.6	10.5
3% LIG	34.6	25.6	9.0

**Table 2:** Effective friction angle and cohesion of pure soil and LIG-treated soil.

Soil Reference	Effective Cohesion, c′ (kPa)	Effective Internal Friction Angle, ϕ′ (°)
Pure soil	24	28.9
1% LIG	38	24.0
3% LIG	53	22.5

**Table 3:** Values of reduction factor and difference in reduction factor during each wetting/drying cycle.

Cycle	*R_f_* (100%)			Cycles	∆*R_f_*		
	Pure Soil	1% LIG	3% LIG		Pure Soil	1% LIG	3% LIG
0	0.0	0.0	0.0	0–1	19.0	50.1	30.3
1	19.0	50.1	30.3	1–2	3.2	5.3	13.1
2	22.2	55.4	43.4	2–3	16.3	4.8	13.0
3	38.5	60.2	56.4	3–4	0.3	1.6	−1.0
4	38.9	61.8	55.4	4–5	7.8	3.6	8.6
5	46.7	65.4	63.9	5–6	6.4	−0.7	1.1
6	53.1	64.7	65.0				

## Data Availability

The data presented in this study are available on request from the corresponding author.
